# Supplementary material for: Analysis of PPARγ Signaling Activity in Psoriasis
Source: Int J Mol Sci. 2021 Aug 10;22(16):8603. doi: 10.3390/ijms22168603 (PMC8395241; doi:10.3390/ijms22168603)
Supplement: Supplementary file 1 [file ijms-22-08603-s001.zip › Supplemental materials_Analysis of PPARg signaling activity in psoriasis/Pathway models/Models images and html files/PPARG inhibits phenotypes assotiated with psoriasis/118746.html]

hypertension


# Disease hypertension

|  |  |
| --- | --- |
| URN | urn:agi-meshdis:Hypertension |
| Total Entities | 29 |
| Connectivity | 11285 |
| Name | hypertension |

---

|  |  |
| --- | --- |
| ChildConcepts | transient hypertension of pregnancy |
|  | Liddle's syndrome |
|  | pulmonary hypertension |
|  | diabetic hypertension |
|  | pregnancy-induced hypertension |
|  | essential hypertension |
|  | distal arthrogryposis type 3 |
|  | genetic hypertension |
|  | hypertensive crisis |
|  | orthostatic hypertension |
|  | systolic hypertension |
|  | white coat hypertension |
|  | masked hypertension |
|  | resistant hypertension |
|  | dyslipidemic hypertension |
|  | portal hypertension |
|  | experimental hypertension |
|  | malignant hypertension |
|  | renovascular hypertension |
|  | ocular hypertension |
|  | intracranial hypertension |
|  | metabolic syndrome X |
|  | apparent mineralocorticoid excess syndrome |
|  | prehypertension |
|  | borderline hypertension |
|  | intra-abdominal hypertension |
|  | idiopathic and/or familial pulmonary arterial hypertension |
|  | hypertensive heart disease |
|  | ocular ischemic syndrome |

---

|  |  |
| --- | --- |
| Pathway | Proteins with Altered Expression in Aging |
|  | Proteins Involved in Arterial Hypertension |
|  | Renin-Angiotensin-Aldosterone System in Myocardial ischemia |
|  | Ritonavir Induced Cardiovascular Dysfunction |
|  | PPARG inhibits phenotypes assotiated with psoriasis |
|  | Neighbors of hypertension |
|  | Overview. Diseases of circulatory system (chapter 8) |

---

|  |  |
| --- | --- |
| MedScan ID | 9010369 |

---

|  |  |
| --- | --- |
| Alias | high renin hypertension |
|  | hypertensions in acute |
|  | Hypertensive Disorder |
|  | neurogenic hypertension |
|  | Hypertensive Disorders |
|  | neurogenic hypertensions |
|  | Hypertension Arterial |
|  | controlled hypertension |
|  | Vascular Hypertension |
|  | cardiovascular hypertension |
|  | hypertensive patients |
|  | salt high blood pressure |
|  | Hypertensive Vascular Degeneration |
|  | Hypertensions |
|  | hyper-tensive disorders |
|  | hypertensive response |
|  | hyper-tension |
|  | High Blood Pressure Disorders |
|  | hypertension in acute |
|  | Hypertension Arterials |
|  | pre-existent hypertension |
|  | Blood Pressures, High |
|  | salt hypertension |
|  | acute hypertensions |
|  | endocrine hypertension |
|  | Hypertensive Vascular Diseases |
|  | Blood Pressure, High |
|  | arterial hypertension |
|  | arterial hypertensions |
|  | acute hypertension |
|  | preexistent hypertension |
|  | Hypertensive Diseases |
|  | secondary hypertension |
|  | HTN |
|  | Hypertensive events |
|  | Hyperpiesis |
|  | Hypertensive Vascular Disease |
|  | Pressure, High Blood |
|  | Hypertensive Disease |
|  | Hypertension |
|  | hypertensives |
|  | responses in hypertensive |
|  | Systemic Hypertension |
|  | High Blood Pressure |
|  | Systemic Arterial Hypertension |
|  | secondary hypertensions |
|  | refractory hypertension |
|  | High Blood Pressures |
|  | Hyperpiesia |
|  | hypertensive responses |
|  | Vascular Hypertensive Disorders |

---

|  |  |
| --- | --- |
| MeSH ID | D006973 |
|  | M0010859 |

---

|  |  |
| --- | --- |
| MeSH Heading | Hypertension |

---
